# Family-based intervention in adolescent restrictive eating disorders: early treatment response and low weight suppression is associated with favourable one-year outcome

**DOI:** 10.1186/s12888-017-1486-9

**Published:** 2017-09-15

**Authors:** Ingemar Swenne, Thomas Parling, Helena Salonen Ros

**Affiliations:** 1 Department of Women’s and Children’s Health, Uppsala University, S-75185 Uppsala, Sweden; 2 Department of Psychology, Uppsala University, Uppsala, Sweden; 3 Centre for Psychiatry Research, Department of Clinical Neuroscience, Karolinska institutet & Stockholm Health Care Services, Stockholm County Council, Stockholm, Sweden; 4 Department of Neuroscience, Child and Adolescent Psychiatry, Uppsala University, Uppsala, Sweden

**Keywords:** Adolescents, Anorexia nervosa, Eating disorder, Family-based intervention, Weight suppression, Early treatment response

## Abstract

**Background:**

Family-based treatments are first-line treatments for adolescents with restrictive eating disorders (ED) but have to be improved since outcome is poor for some. We have investigated the one-year outcome of a family-based intervention programme with defined and decisive interventions at the start of treatment.

**Method:**

Data pertaining 201 adolescents with restrictive ED with features of anorexia nervosa but not fulfilling the weight criterion starting treatment 2010-2015, had a wide range of body mass index (BMI) and of weight loss at presentation, and completed a one-year follow-up was analysed. Recovery from the ED was defined as an Eating Disorder Examination-questionnaire (EDE-Q) score < 2.0 or as not fulfilling criteria for an ED at a clinical interview.

**Results:**

By EDE-Q 130 (65%) had recovered at 1 year and by clinical interview 106 (53%). According to the EDE-Q criterion recovery was independently associated with lower EDE-Q score at presentation, higher weight gain after 3 months of treatment and lower weight suppression at follow-up, weight suppression being defined as the difference between premorbid and current BMI. Not fulfilling criteria for an ED was associated with the same factors and also by higher BMI at presentation.

**Conclusion:**

The observations that low weight and high ED cognitions confer a poor prognosis but that rapid weight gain at the start of treatment predicts a better prognosis are presently extended to adolescents with restrictive ED with a wide range of BMI at presentation. High weight suppression at follow-up is associated with a poor prognosis and indicates the importance of taking premorbid BMI into account when setting weight targets for treatment.

## Background

In anorexia nervosa (AN) and other restrictive eating disorders (other specified feeding and eating disorders – restrictive subtype; OSFEDr) with adolescent onset treatment outcome has to be improved since some patients develop a chronic course of the disease and even excess mortality later in life. Family interventions are proposed as first-line treatments [1, 2] and the evidence-base for family-based treatment (FBT) of AN is increasing [3, 4]. Family-based interventions are out-patient treatments which emphasize the role of the parents. At the start of treatment, they should be supported to take charge of meal routines, help their adolescent to normalize eating and initiate weight gain.

In FBT of AN weight gain is essential and mediates psychological recovery [5]. Rapid weight gain during the initial phase of FBT is associated with favourable long-term outcome in terms of normalisation and maintenance of weight and of recovery from eating disordered (ED) psychopathology [6–9]. In AN there is by definition underweight and it stands to reason that weight gain is needed. In OSFEDr the adolescent may at presentation be near normal weight. This may be so if weight loss is small but could also be the result of a greater weight loss starting from an overweight [10, 11]. These adolescents all have to normalise eating behaviours but treatment goals in terms of weight gain may be subject to dispute and depend on how target weights are set [12–14]. This is a relevant problem since a considerable proportion of adolescents with restrictive ED start weight loss from a weight or body mass index (BMI) above population average and are not severely underweight at presentation [10, 11, 15, 16].

The difference between premorbid and current weight has been termed weight suppression [17]. In women with bulimia nervosa maintenance of weight suppression during treatment predicts a less favourable outcome [18, 19]. There is recent data to suggest that this may also be the case in AN [20–23]. Weight suppression adds a hitherto not often considered aspect of weight recovery. Recovering weight to attain a target based on a standard for gender, age and stature may underestimate a target taking in consideration individual premorbid weight/BMI.

We have recently shown that decisive parental management at the start of treatment of adolescents with restrictive ED is effective in promoting early rapid weight gain and decreasing ED psychopathology as early as at a 3-month follow-up [24]. It is notable that the majority of the adolescents in this study had a restrictive ED but did not fulfil weight criteria for AN; i.e. many were despite weight loss near normal weight. Weight gain was nevertheless paralleled by a decrease in ED symptoms. This indicates that normalisation of eating behaviours and weight gain promotes recovery also in adolescents with ED who has not reached the very low weight of AN. We have now extended this study to a one-year follow-up of adolescents with OSFEDr, i.e. with an ED with features of AN but not reaching the low weight of AN. In doing so, we have included a larger number of adolescents and also documented data on weight before the onset of the ED.

We hypothesised that early rapid weight gain and absence of weight suppression at the one-year follow-up would be associated with a favourable outcome. We also hypothesised that the preliminary results of our previous study [24] would be confirmed in a larger cohort, i.e. the early interventions performed by the parents would be reflected in a favourable outcome.

## Methods

### Participants

The Eating Disorder Unit (EDU) at the Department of Child and Adolescent Psychiatry (CAP) of the Uppsala University Hospital is a specialized unit offering treatment to all patients with an ED and <18 years of age in the county (population 335,882 of which 69,314 < 18 years on Dec 31 2010). During the period Aug 2010 – July 2015 352 patients from the catchment area, previously not treated for an ED, were assessed and offered a start of treatment at the EDU. Three girls who were close to 18 years of age were transferred to an adult psychiatric service following assessment and were not included. Eleven adolescents with (subthreshold) bulimia nervosa were not included in the study. Eleven adolescents and their families declined treatment and three accessed treatment elsewhere following assessment. Eligible for follow-up were 41 adolescents with AN and 289 with OSFEDr. The cut-off for the weight criterion was a body mass index standard deviation score (BMI SDS) of −2,00 [25]. Only those with OSFEDr were included in the present study.

### Procedure

The initial assessment of new patients was performed by a paediatrician and followed a structured protocol including the history of the ED, demographic and medical history including medical and psychiatric comorbidity, a physical examination with measurements of weight and height, and blood sampling. The history of weight and height changes was obtained from growth charts procured from the school health services. An ED diagnosis was established according to the Diagnostic and Statistical Manual of Mental Disorders (DSM-IV).

At the initial assessment, parents were informed that start of treatment aims at normalising eating by their taking charge of meal routines and stopping weight loss. Parents received recommendations on what constitutes a normal meal and were suggested to serve three main meals and two snacks every day. They were also advised on how to handle meal-related anxiety and the urge for compensatory behaviours. They were clearly advised on four points: 1) Attending school is advised against as long as the adolescent cannot handle meals on her/his own, 2) All meals should be eaten together with a parent, 3) All exercise is banned and 4) The adolescent should be prevented from vomiting. The advice given was similar irrespective of the weight and weight loss of the adolescent.

The first follow-up at the EDU was 1 week (7.8 ± 2.0 days, range 5-17 days) after the initial assessment. Patients were weighed and the first week of treatment was reviewed. Based on whether the four above recommendations had been accomplished patients were categorised as “accomplishers” (all four points accomplished) or “non-accomplishers” (at least one point not accomplished). Note that the amount of food eaten did not influence accomplishment status. The categorisation into accomplishers/non-accomplishers used in the present analyses thus refers only to the accomplishments during the first week following initial assessment. Self-report questionnaires were administered to assess ED and depressive symptoms. Families received further information on the treatment programme at the EDU and decisions on treatment and follow-up were according to clinical routines and the treatment programme of the EDU. This could now involve new decisions on school attendance, meals, and exercise.

Weight gain at 1 week, 1 month and 3 months after initial assessment was calculated. When weight was not measured at the exact time interval, weight changes were corrected to 7-day, 30-day and 13-week periods, respectively.

One year after start of treatment a face-to-face follow-up was attended by 201 (70% of those assessed, 73% of those starting treatment) of the patients. This was usually performed by the therapist who had seen the patient/family for the past year. The patients’ records were scrutinized for new comorbid diagnoses. The follow-up visit included measurement of weight and length and administration of the self-report questionnaires used at assessment. A clinical interview to map ED ideation and ED behaviours such as food restriction, vomiting or exercising for weight control was performed to assess whether the adolescents fulfilled criteria for an ED.

All participants and their guardians consented to participate in the study. The protocol was approved by the Ethics committee of the Faculty of Medicine of Uppsala University.

### Study measures and statistics

BMI was calculated as weight/height^2^ (kg/m^2^) and recalculated into BMI SDS, which constitutes a measure of leanness corrected for age and height [26]. At assessment weight loss was calculated as the difference between weight at presentation and the highest recorded premorbid weight. Weight suppression was calculated as the difference between BMI SDS at presentation/follow-up and BMI SDS at the highest premorbid weight.

The self-report instruments Eating Disorders Examination-Questionnaire youth version (EDE-Q) [27] and Montgomery-Åsberg Depression Rating Scale-Self report (MADRS-S) [28] were used to assess ED and depressive symptoms, respectively.

ED diagnoses were according to DSM-IV criteria but retrospectively recoded into DSM-5 criteria. BMI SDS < −2.0 was used as the weight criterion for AN [25]. In young individuals, ED symptoms may not be verbally expressed. Behaviours may nevertheless indicate food avoidance and preoccupation/dissatisfaction with weight and shape. Care was therefore taken to assess both symptoms verbalized by the adolescents and behaviours reported by their parents [29, 30]. Depression was diagnosed according to DSM-IV criteria for a “depressive episode”. “Self-destructive behaviours” included cutting, burning and intoxication with prescription drugs. “Neuropsychiatric diagnoses” at the one-year follow-up includes obsessive compulsive disorder (OCD), attention deficit/hyperactivity disorder (ADHD), attention deficit disorder (ADD), Tourette’s syndrome and autism spectrum disorders and comprises diagnoses present at presentation of the ED, new diagnoses during the first year of treatment of the ED and also if assessment for such a diagnosis had been suggested but not yet initiated or completed.

Two measures of recovery were used: 1) EDE-Q global score < 2.0, which corresponds to a value at or below the mean + 1 SD of the score of adolescent community samples [26, 31–33] and to the cut-off for a clinical significant score in Swedish samples [34] and 2) not fulfilling diagnostic criteria for an ED at the follow-up interview.

Statistical analyses were performed using SPSS 20.0.0. Values are given as means ± SD. Differences in weight and psychometric measures were first compared using Student’s t-test for independent samples or Chi-square test for continuous or categorical data, respectively. A logistic regression analysis was used to further analyse outcome.

### Treatment

Treatment is provided in an outpatient setting, and reinforced with day-care when needed [25]. Day-care is for brief periods, usually only 1 or 2 weeks, when the adolescents have meals together with their parents and the nursing staff at the EDU. The object is then not primarily to achieve refeeding and weight gain but rather to support parents to take command of meals and transfer this ability to home. In-patient treatment is not part of the treatment programme and is used only as an emergency measure. Nursing staff and psychologists are trained in cognitive behavioural therapy (CBT) and FBT. Treatment is based on family interventions and strongly emphasises parental involvement in the care of the child. In this context, an important feature of the Swedish social security system is the possibility for reimbursed parental leave to care for a severely sick child up to the age of 18 and for as long a period as needed.

The first step of the treatment programme has the single focus of stopping on-going weight loss and normalising routines around meals. This is emphasised already at the initial assessment as described above [24]. The second step of the programme ensues when eating has been almost normalised and routines of everyday life are re-established. It aims at restoring weight at a rate of 0.5-1 kg/week, regardless of the ED diagnosis. If weight gain is slow and/or weight deficit large, nutritional supplements may be introduced. A final step starts with a gradual reintroduction into school. This begins when eating has been normalised (although meal support may still be necessary), a substantial proportion of weight deficit has been recovered, and vigilance over daily routines can be reduced. Remaining difficulties, such as low self-esteem, over-evaluation of weight and shape, perfectionism and/or interpersonal difficulties can now be targeted to prevent relapse of the ED. The presence of comorbid psychiatric disease may have to be reassessed and the need for support in areas outside the core features of the ED considered. The treatment programme does not have a fixed duration or a fixed number of sessions but is goal oriented. Treatment is offered as long as any lingering features of the ED are present.

The structure and content of the treatment is strongly influenced by FBT [35]. The present approach differs from that of FBT in that the parents are suggested interventions at the first session rather than being empowered to find for themselves which actions to take. It also differs in that CBT is used for remaining difficulties with the ED and for comorbid disorders. The approach resembles FBT in that it emphasizes that it is the parents who should re-establish family routines, and although advised on interventions, would have to be empowered to solve different problems which arise during treatment.

Pharmacological treatment, usually selective serotonin reuptake inhibitors (SSRI), is used for comorbid disorders such as depression and OCD following normalisation of meal routines and initiation of weight gain. For anxiety and insomnia promethazine is tried first. Olanzapin is used for severe anxiety, rumination over weight and shape, and for excessive urges to exercise. Benzodiazepines are seldom used.

## Results

### Characteristics of patients at presentation

Characteristics of the 201 patients with complete data including the one-year follow-up are given in Table 1. At their highest premorbid weight, the patients were less lean than the population average as evidenced by a BMI SDS above zero (*p* < 0.001). Duration of disease was on average less than a year but weight loss was varying (range -5.0 - 27.4 kg). Exercise for weight control was common but vomiting was reported by only one fifth. EDE-Q global score was high but varying (range 0-5.8).

**Table 1:** Characteristics at presentation of 201 adolescents with restrictive eating disorders with features of anorexia nervosa but not fulfilling the weight criterion

Age at top weight (years)	14.1 ± 1.7 (range 7.6 – 17.3)
BMI SDS at top weight	0.69 ± 1.00 (range - 1.22 – 3.11)
Gender (M/F)	13/201
Age at presentation (years)	15.0 ± 1.7 (range 9.4 – 17.8)
Duration of ED symptoms (months)	9.6 ± 8.7 (range 2 – 60)
BMI SDS at presentation	−0.56 ± 1.04 (range - 1.96 – 2.46)
Weight loss (kg)	6.3 ± 5.7 (range - 5.0 – 27.4)
Menstrual status (premenarcheal/secondary amenorrhea/no amenorrhea/hormonal anticonception)	17/54/103/14 (9/29/55/7%)
Exercise for weight control	139 (69%)
Vomiting for weight control	39 (19%)
Self-destructive behaviour	23 (11%)
EDE-Q global score	2.9 ± 1.7
MADRS-S	18 ± 11
Depression	43 (21%)
Neuropsychiatric diagnoses	9 (4%)

Values are means ± standard deviations

*BMI* body mass index, *SDS* standard deviation score, *EDE-Q* eating disorders examination-questionnaire, *MADRS-S* Montgomery-Åsberg depression rating scale-self report

Forty-eight patients (24%) had had previous contacts with CAP, in most cases for depressive and/or anxiety disorders. Psychiatric comorbidity, diagnosed before assessment of the ED, also included ADHD/ADD (8), OCD (1) and posttraumatic stress disorder (1). Psychotropic medication at assessment included SSRI (7 patients), central stimulants (6), prometazin (2) and melatonin (2).

Somatic comorbidity included coeliac disease (5 patients), type 1 diabetes (3), hypothyreosis (2), growth hormone deficiency (1), inflammatory bowel disease (1), limb deformity (1), Langerhans histiocytosis (1), kidney failure (1), juvenile idiopathic arthritis (1) and pubertas praecox (1).

### Characteristics of patients not followed up

Eighty-eight (30%) of the assessed patients did not take part in the one-year face-to-face follow-up. Ten declined treatment and three had moved from the catchment area. Six were transferred to other CAP units because of neuropsychiatric disorders or a complex picture including self-harming behaviours. Fifteen reported that they were well after only a few sessions, discontinued treatment and ended all contact with the EDU. One patient was missed for follow-up and the remaining patients declined a follow-up interview.

In comparison with patients who attended the one-year follow-up the non-attenders did not differ at presentation with respect to gender, age, BMI SDS, duration of disease, weight loss, weight suppression, menstrual status, EDE-Q global score, MADRS-S score or the use of exercise to control weight (*p* > 0.05). They differed in that the non-attenders reported a higher use of vomiting to control weight (40 versus 19% in attenders, *p* < 0.001) and that reported self-destructive behaviours were more common (27 versus 11%, *p* < 0.01) compared to attenders. The prevalence of depression (17 versus 21%, *p* > 0.05) did not differ. Of the patients who did not attend the one-year follow-up data on weight gain during the start of treatment was available for 75 (85%). The one-week, one-month and three-month weight gains did not differ from those followed up. Data on weight gain, BMI SDS and weight suppression at approximately 1 year was available for 52 (59%) non-attenders and did not differ from that of the attenders. However, comorbid neuropsychiatric disease was more prevalent in non-attenders (29 versus 15%, *p* = 0.031).

### Treatment outcome

Treatment outcome was evaluated for the 201 patients who had been followed up at 1 year with an interview and self-report instruments. The majority of patients had been treated as out-patients. Twenty-four (12%) had received day-care for some period. There had been six emergency hospitalisations of four (2%) different patients to a paediatric ward due to refusal to eat, progressive weight loss and medical instability. Three of these patients had been tube fed. There had been six hospitalisations of six (3%) different patients to a CAP ward due to depressive symptoms and suicidal ideation. Following discharge patients returned to out-patient treatment/day care.

During some part of the first year of treatment six (3%) patients had been treated with benzodiazepines, 16 (8%) with olanzapine, 41 (20%) with SSRI and 43 (21%) with other psychotropic drugs including central stimulants for neuropsychiatric disorders and promethazine or melatonin for insomnia. At the one-year follow-up one (0.5%) patient was on benzodiazepines, six (3%) on olanzapine, 38 (19%) on SSRI and 23 (11%) on other drugs.

When an EDE-Q score < 2.0 was used as an indicator of recovery at follow-up, 130 patients (65%) fulfilled the criterion (Table 2). At presentation, these patients differed from those not recovered in that they had lower BMI SDS, lower EDE-Q global and MADRS-S scores, and a higher proportion of males. They did not differ with respect to age, duration of ED symptoms, weight loss, the use of exercise or vomiting for weight control, self-destructive behaviours or the prevalence of depression or diagnosed neuropsychiatric disorders. At the start of treatment recovered patients had more rapid weight gain for the first 3 months but there was no difference in accomplishment of the initial interventions. At the one-year follow-up they had a greater weight gain, less weight suppression, and a lower prevalence of depression and neuropsychiatric diagnoses. A substantial proportion (18%) of the patients with EDE-Q global score < 2,0 at follow-up were, however, judged to have an ED at the follow-up interview.

**Table 2:** One-year follow-up of family-based treatment of 201 adolescents with restrictive eating disorders with features of anorexia nervosa but not fulfilling the weight criterion

	EDE-Q global score at 1-year follow-up		Eating disorder at 1-year follow-up	
	<2.0	≥2.0	No eating disorder	Persisting eating disorder
n	130	71	106	95
Premorbid				
Age at top weight (years)	14.1 ± 1.7	14.2 ± 1.8	14.2 ± 1.6	14.0 ± 1.8
BMISDS at top weight	0.55 ± 1.03	1.03 ± 0.88**	0.63 ± 0.93	0.76 ± 1.08
Presentation				
Gender (n; M/F)	12/118	1/71*	11/96	2/93*
Age (years)	14.9 ± 1.6	15.2 ± 1.8	15.0 ± 1.6	15.0 ± 1.8
Duration of ED symptoms (months)	9.5 ± 8.4	9.9 ± 9.3	10.6 ± 9.2	8.5 ± 8.0
BMISDS	−0.69 ± 1.10	−0.32 ± 0.88*	−0.56 ± 1.08	−0.57 ± 0.99
Weight loss (kg)	5.9 ± 5.2	7.2 ± 6.3	5.8 ± 5.4	6.9 ± 5.9
Weight suppression (SDS)	1.25 ± 0.86	1.33 ± 0.87	1.20 ± 0.90	1.37 ± 0.81
Exercise for weight control (n)	91 (70%)	48 (68%)	72 (68%)	67 (71%)
Vomiting for weight control (n)	23 (18%)	16 (23%)	21 (20%)	18 (19%)
Self-destructive behaviour (n)	15 (12%)	8 (11%)	11 (10%)	12 (13%)
EDE-Q global score	2.6 ± 1.7	3.6 ± 1.6***	2.6 ± 1.6	3.2 ± 1.7*
MADRS-S	16 ± 10	22 ± 11***	16 ± 10	20 ± 11*
Depression (n)	23 (18%)	20 (28%)	18 (17%)	25 (26%)
Neuropsychiatric diagnoses (n)	6 (5%)	3 (4%)	6 (5%)	3 (3%)
Start of treatment				
Parents accomplishing interventions during 1st week (n)	95/127 (75%)	41/65 (63%)	74/103 (72%)	62/89 (70%)
1-week weight gain (kg)	0.6 ± 1.0	0.3 ± 0.8*	0.7 ± 1.1	0.4 ± 0.7*
1-month weight gain (kg)	1.8 ± 1.8	0.6 ± 1.7***	1.9 ± 1.8	0.8 ± 1.6***
3-months weight gain (kg)	4.0 ± 3.0	1.6 ± 3.2***	4.2 ± 3.1	2.0 ± 3.2***
1-year follow-up				
Age (years)	15.9 ± 1.6	16.3 ± 1.8	16.0 ± 1.6	16.1 ± 1.8
BMISDS	0.06 ± 0.90	0.01 ± 0.96	0.17 ± 0.85	−0.11 ± 0.97*
Weight gain (kg)	6.9 ± 4.6	3.9 ± 6.7***	7.1 ± 4.4	4.5 ± 6.4**
Weight suppression (SDS)	0.50 ± 0.83	0.95 ± 0.95**	0.46 ± 0.74	0.88 ± 1.01***
EDE-Q global score	0.7 ± 0.6	3.5 ± 1.0***	0.7 ± 0.7	2.8 ± 1.5***
ED diagnoses (no ED/AN/BN/OSFEDr)	102/1/1/26 (78/1/1/20%)	4/3/2/62 (6/4/3/87%)***	125/0/0/0 (100/0/0/0%)	0/4/3/88 (0/4/3/93%)***
MADRS-S	7 ± 7	21 ± 10***	7 ± 8	17 ± 11***
Depression (n)	8 (6%)	24 (34%)***	7 (7%)	25 (27%)***
Neuropsychiatric diagnoses (n)	13 (10%)	17 (24%)*	12 (11%)	19 (20%)*

Values are means ± standard deviations. Significance of difference between recovered adolescents (EDE-Q < 2.0 or absence of an ED) and those with a persisting disease: *p < 0.05, **p < 0.01, ***p < 0.001 by Student’s t-test for continuous data and Chi-square test for categorical data

*ED* eating disorder, *BMI* body mass index, *SDS* standard deviation score, *EDE-Q* eating disorders examination-questionnaire, *MADRS-S* Montgomery-Åsberg depression rating scale-self report, *AN* anorexia nervosa, *BN* (subthreshold) bulimia nervosa, *OSFEDr* other specified feeding and eating disorders-restrictive subtype

One hundred and six (53%) patients did not fulfil diagnostic criteria for an ED at the one-year follow-up (Table 2). At presentation these patients differed from those who still had an ED in that they had lower EDE-Q global and MADRS-S scores and a higher proportion of males. They did not differ with respect to age, duration of ED symptoms, weight loss, the use of exercise or vomiting for weight control, self-destructive behaviours or the prevalence of depression or diagnosed neuropsychiatric disorders. At start of treatment recovered patients had a greater weight gain for the first 3 months although there was no difference in accomplishment of the initial interventions. At the one-year follow-up they had higher BMI SDS, greater weight gain, less weight suppression, and a lower prevalence of depression and neuropsychiatric diagnoses.

### Predictors of outcome

Low weight, high ED cognitions and psychiatric comorbidity are predictors of poor outcome in AN [36–38]. Therefore, one-unit changes of BMI SDS at presentation, EDE-Q global score at presentation and the presence of comorbid neuropsychiatric disease together with weight suppression at follow-up and a 3 kg weight gain at 3 months were analysed against the outcome measures in a logistic regression analysis (Table 3).

**Table 3:** Prediction of one-year outcome of family-based treatment of 201 adolescents with restrictive eating disorders with features of anorexia nervosa but not fulfilling the weight criterion

Outcome	Predictor	Odds ratio	95% CI	p
EDE-Q global score < 2.0	BMI SDS at presentation	1.11	0.72–1.71	NS
	EDE-Q global score at presentation	0.66	0.52–0.84	0.001
	3-month weight gain	1.26	1.09–1.46	0.002
	Weight suppression at follow-up	0.57	0.36–0.89	0.013
	Neuropsychiatric disease	0.47	0.18–1.22	NS
No eating disorder	BMI SDS at presentation	1.78	1.18–2.69	0.006
	EDE-Q global score at presentation	0.74	0.60–0.92	0.007
	3-month weight gain	1.30	1.14–1.49	0.0001
	Weight suppression at follow-up	0.61	0.40–0.94	0.023
	Neuropsychiatric disease	0.61	0.24–1.55	NS

*BMI* body mass index, *SDS* standard deviation score, *EDE-Q* eating disorders examination-questionnaire, *CI* confidence interval

An EDE-Q global score < 2.0 was predicted by lower EDE-Q global scores at presentation, by higher weight gain at 3 months and associated with lower weight suppression at follow-up. Not fulfilling criteria for an ED at the follow-up interview was associated with the same factors and also predicted by higher BMI at presentation. Adding age, premorbid BMI SDS, duration of the ED, weight loss, rate of weight loss, behaviours used for weight control, MADRS-S score at presentation, depression at presentation, reporting self-destructive behaviour at presentation, accomplishment of interventions at the start of treatment, weight gain at follow-up, depression at follow-up or self-destructive behaviour at follow-up to the models did not improve prediction.

## Discussion

This study shows that higher BMI and lower ED cognitions at presentation, rapid weight gain at the start of treatment and lower weight suppression at follow-up are associated with a favourable one-year outcome following a family-based intervention in adolescents with restrictive ED with a wide range of BMI at presentation.

When assessing recovery from AN, a combination of weight recovery and remission of ED cognitions is commonly used [39]. Weight recovery is then defined by a standard for gender, age and height. In the present OSFEDr population such a weight criterion is not applicable since a large proportion of the patients would fulfil it already at presentation, despite weight loss [11]. The definition of recovery from the ED therefore had to resort to a clinical interview and self-reported symptoms. These two definitions of favourable outcome differed in that absence of an ED but not the global EDE-Q cut-off score (<2) was associated with BMI SDS at presentation. This is explained by that the patients who had a low global EDE-Q score but nevertheless were judged to have and ED at the interview had lower BMI SDS at presentation.

Low BMI and high ED psychopathology at presentation are predictors of poor outcome of AN in a number of studies [36–38]. The present study now extends this observation to the wider weight range of an OSFEDr population. Long duration of the ED, higher age at presentation and vomiting/purging are also common predictors of poor outcome of AN but are not replicated in our sample. It may be that the present limited spans of duration of disease and of age precludes differentiation. Vomiting is registered as present or absent and a poor prognosis may be predicted only by frequent vomiting.

The prediction of favourable outcome of AN by rapid early weight gain [40] is applicable also in the present OSFEDr population. We have previously shown that the weight gain at start of treatment is promoted by a consistent intervention of the parents [24]. The effect of the interventions of the parents to promote early weight gain is now extended to predict a favourable overall outcome of the ED across a wide BMI range. This may be so because it is an indicator of the parents’ successful interventions against ED behaviours and their establishment of normal meal routines. As a result, the switch from negative energy balance to eating inevitably will lead to weight gain irrespective of the BMI. The association of early weight gain with favourable outcome may also be related to that many patients, despite near-normal weight at presentation, have to gain weight to recover their premorbid weight/BMI trajectory. This was indicated by that high weight suppression was independently associated with persistence of the ED. Weight recovery thus needs to be aimed at the premorbid growth trajectory of the adolescent rather than at a weight standardized for gender, age, and height.

The association of weight suppression with persisting ED can be understood if the consequences of negative energy balance and weight reduction are considered. During weight loss energy metabolism change to promote weight (re) gain and there are changes in circulating hormones which increase appetite and favour energy storage [41–43]. Such adaptations persist when a stable weight is maintained below a previous, higher weight [41–43]. To maintain such a suppressed weight a life style including exercise, cognitive restriction of food intake and self-monitoring of weight is necessary [41–44], behaviours which would be in contraposition with the treatment of an ED. This would be resolved if the weight target is set at a level where constant vigilance over weight would not be necessary.

The necessity to return to the premorbid growth trajectory conforms to the concept of canalization of growth and development. Canalization implies a genetically determined buffering against temporary perturbations, which sustains development along a predetermined trajectory. The BMI trajectory of healthy adolescents [45] would be an example of such canalization and there is also evidence of such canalization in adolescents with AN [46]. In restrictive ED return of menstruations [47, 48] and resumption of linear growth [49] are associated with a return to the premorbid growth trajectory. These observations of developmental canalisation are now extended to that recovery from an ED is associated with the recovery of the premorbid weight/BMI trajectory. However, if the premorbid trajectory is in the very obese range such a treatment goal would not be medically sound but has to be reduced. For such obese patients it is nevertheless important to appreciate that a goal of an average weight for height and age is too low.

Psychiatric comorbidity is in many studies a predictor of poor outcome [37, 38]. Presently, neuropsychiatric disorders were more prevalent in those not recovered but was not independently associated with poor outcome. This would suggest that it was possible to handle the ED in the present one-year perspective. The comorbid psychiatric diagnoses may influence other aspects of daily life and the patients would therefore benefit from other treatments and supportive measures once the ED has been addressed. Conclusions on the effects of such comorbidity on the outcome should, however, be cautious since psychiatric comorbidity was over-represented in those not followed up.

Depression and self-reported depressive symptoms were more prevalent in those not recovered but were not independently associated with poor outcome. Symptoms such as low self-esteem, a sense of inadequacy and poor interpersonal relationships are common in both depression and ED. In ED such symptoms are related to the degree of ED cognitions [50]. Symptoms such as low energy, inability to concentrate and sleep disturbances may be explained by starvation [51]. All these seemingly depression-related symptoms improve during refeeding of an ED [52–54]. Since they improve in parallel with the ED they need not be targeted at the start of treatment [35].

This study confirms previous observations of that low weight and a high level of ED cognitions are associated with unfavourable outcome. It has, however, further clinical implications since it identifies potentially modifiable predictors of outcome. Weight loss and entrenchment in ED cognitions can be reduced if an ED specialist service is easily accessible and start of treatment is without delay. The chain of referral and the administrative efficiency of the service organisation may thus influence treatment outcome. The importance of the early treatment response for the long-term outcome indicates that much effort should be put into assessment and start of treatment, which indeed can be performed simultaneously. In family-based treatments this means that parents should be intensely coached to help their adolescents by immediately taking over and normalizing meal routines. Our data also indicate that setting weight targets should take into account premorbid weight/BMI.

The study has a short-coming in that not all patients were followed up. It is, however, notable that those not followed up in most respects did not differ from the sample examined. A caveat is that vomiting to control weight, self-destructive behaviours and neuropsychiatric disorders were more prevalent among those not followed up. Another problem may be the use of EDE-Q rather than the EDE interview. Although there is a general agreement between the two formats [55] differences may emerge. This is indicated by the group of patients who scored low on the EDE-Q but nevertheless were judged to have an ED at the interview. It should be noted that the therapists performing the interviews could be biased since they were not blinded to the treatment given or the course of the disease. On the other hand, knowing the patient may help to disclose details of the ED, which otherwise would pass undetected. It is furthermore necessary to extend the one-year follow-up to confirm the results in the long-term. It is then promising to note that the one-year outcome of family-based treatment is stable over longer periods [9].

The study has a strength in that adolescents with a wide range of BMI at presentation were examined. Most of them represent the large group of patients categorised as OSFED. Despite higher BMI they may be medically compromised by weight loss and have ED cognitions as strong as patients with AN [10, 11, 56] and should not be considered to have a “mild” type of ED. There are further strengths in that the initial sample represents almost all patients in the catchment area, are treated at a single specialist service and according to a uniform protocol [25]. The availability of objective measures of premorbid weight/BMI further adds to the strength of the study.

## Conclusion

The present outcome with more than half of the adolescents with OSFEDr in remission at a one-year follow-up compares well with the results obtained with FBT for AN [7]. Patients are, however, difficult to compare. In our patients, to recover from severe underweight is the challenge for only a minority. In patients with OSFEDr the difficulty to motivate and support parents and adolescents to normalize eating and recover a personal weight trajectory above population average may be as challenging, especially since ED cognitions may be as strong as for their very low-weight peers. The present investigation nevertheless indicates that also these patients benefit from a decisive start of treatment with rapid weight gain.

## Abbreviations

ADD: Attention deficit disorder
ADHD: Attention deficit hyperactivity disorder
AN: Anorexia nervosa
BMI: Body mass index
CAP: Child and adolescent psychiatry
CBT: Cognitive behavioural therapy
ED: Eating disorder
EDE: Eating diorders examination
EDE: Eating disorders unit
EDE-Q: Eating disorders examination – questionnaire
FBT: Family-based therapy
MADRS-S: Montgomery-Åsberg depression rating scale – self report
OCD: Obsessive compulsive disorder
OSFEDr: Other specified feeding and eating disorders – restrictive subtype
SDS: Standard deviation score
SSRI: Selective serotonin uptake inhibitors
